# Amelioration of obesity-related metabolic disorders via supplementation of *Caulerpa lentillifera* in rats fed with a high-fat and high-cholesterol diet

**DOI:** 10.3389/fnut.2022.1010867

**Published:** 2022-09-15

**Authors:** Jeanette Irene Christiene Manoppo, Fahrul Nurkolis, Adriyan Pramono, Martha Ardiaria, Etisa Adi Murbawani, Muhammad Yusuf, Faqrizal Ria Qhabibi, Vincentius Mario Yusuf, Nasim Amar, Muhammad Rico Abdul Karim, Anita Dominique Subali, Hans Natanael, Ronald Rompies, Rifrita Fransisca Halim, Alexander Sam Leonard Bolang, Gregory Joey, Christian Agung Novianto, Happy Kurnia Permatasari

**Affiliations:** ^1^Department of Pediatrics, Sam Ratulangi University/Prof.dr.R.D.Kandou Hospital Manado, Manado, North Sulawesi, Indonesia; ^2^Department of Biological Sciences, Faculty of Sciences and Technology, State Islamic University of Sunan Kalijaga, Yogyakarta, Indonesia; ^3^Department of Nutrition Science, Faculty of Medicine, Universitas Diponegoro, Semarang, Indonesia; ^4^Center of Nutrition Research (CENURE), Universitas Diponegoro, Semarang, Indonesia; ^5^Medical Study Programme, Faculty of Medicine, Brawijaya University, Malang, Indonesia; ^6^Food and Nutrition Department of Sam Ratulangi University, Kampus Unsrat Bahu Street, Manado, Indonesia; ^7^Food Science and Technology Study Programme, Faculty of Agricultural Engineering, IPB University, Bogor, Indonesia; ^8^Department of Biochemistry and Biomolecular, Faculty of Medicine, Brawijaya University, Malang, Indonesia

**Keywords:** *Caulerpa lentillifera*, functional food, lipid profile, PGC-1α, obesity-related metabolic disorders, algae, sea grapes

## Abstract

Dietary modification, including functional foods, could reduce comorbidities due to obesity. An increase in serum glucose and lipids is often seen in obesity. Furthermore, obesity is also characterized by a decrease in antioxidant capacity (i.e., decrease in superoxide dismutase/SOD) and downregulation of peroxisome proliferator-activated receptor γ coactivator-1α (PGC-1α). It has been well established that PGC-1α is important to regulate mitochondrial biogenesis. Sea grapes (*Caulerpa lentillifera*) are known as a traditional food in many Asia-Pacific countries. Recent evidence suggests that sea grapes have many beneficial properties as functional foods and may have potential therapeutic functions. We investigated the effect of sea grapes (*C. lentillifera*) on serum glucose, lipids, PGC-1α, and protein levels of SOD in the liver of *Rattus norvegicus*, which is induced with a high-fat and high-cholesterol diet. A total of four groups were made, each containing ten male *Rattus norvegicus*; group A received a standard dry pellet diet as control, group B received cholesterol- and fat-enriched diets (CFED), groups C and D received CFED and 150 and 450 mg/kg body weight (BW) of sea grape extract, respectively, for 4 weeks. Serum glucose and cholesterol were assessed using a blood auto-analyzer. Serum PGC-1α was measured using ELISA. SOD levels were calculated using the superoxide dismutase assay kit by Sigma-Aldrich with blood taken from liver tissue. In this study, sea grape extracts improved total cholesterol levels better than the CFED and normal groups. The efficacy of total cholesterol improvement was similar between the two doses of sea grape extract. Furthermore, sea grape extract increased PCG-1α levels, especially with the dose of 150 mg/kg BW. Blood glucose was also lower in the groups of sea grape extract. Interestingly, the groups treated with sea grapes extract exhibited higher levels of liver SOD compared to the normal and CFED groups. To conclude, sea grapes (*C. lentillifera*) have promising potential for anti-hyperglycemia and anti-hypercholesterolemia, and for reducing oxidative stress, and providing various health benefits for metabolic disorders.

## Graphical Abstract

Summarized potential mechanisms by which sea grapes may improve metabolic parameters in obesity-related metabolic disorders. 
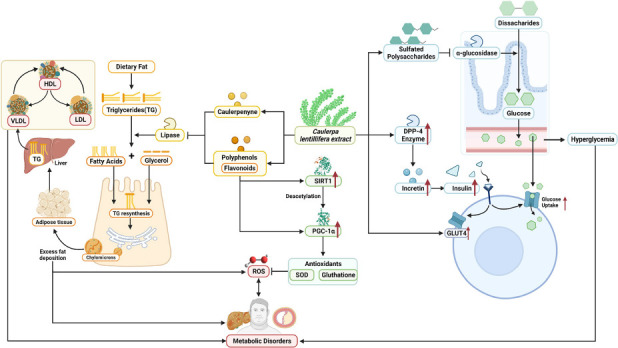

## Introduction

Obesity is a major public health problem that leads to non-communicable diseases such as type 2 diabetes (T2D) and cardiometabolic syndrome (1). It is not only a matter of an increase in body fat within adipose tissue (AT) but more importantly, a decrease in AT function (2). Adipose tissue dysfunction leads to ectopic fat in non-AT tissue, such as the skeletal muscle and the liver (3). More interestingly, fat deposition may cause impairment in glucose and lipid metabolism in the liver (4).

During the development of obesity, oxidative stress can occur in the liver and may partly be determined by the disturbance of mitochondrial function. It occurs partly due to a systematic increase in ROS production and depression of the antioxidant system (5). Studies have demonstrated that an increase in oxidative stress may be associated with a decrease in PGC-1α regulation (6). Importantly, it has been shown that PGC-1α is a major regulator of mitochondrial function and biogenesis (7). In a review, an impairment of ROS regulation within the liver is also associated with the development of non-alcoholic fatty liver disease (8). Collectively, these impairments may also lead to the incidence of metabolic syndrome (i.e., abnormal glucose levels, lipids, and blood pressure).

Maintaining a healthy lifestyle by being physically active and having a balanced diet are still recognized as important to prevent and tackle obesity and metabolic syndrome (9). It has been suggested that nutritious foods are not only from agricultural land but also from the ocean. Ocean areas contain about half of the total global biodiversity, which has many novel and useful compounds (10). One of these biodiversities is sea grapes (*Caulerpa lentillifera*), species in the phylum Chlorophyta and the family Caulerpaceae. This plant is well adapted for mass cultivation in open ponds and is well known for being consumed as a traditional food in many Asia-Pacific countries, including Indonesia (11).

Current research progress shows sea grapes have many beneficial properties that may have the potential for cardiovascular protection and hepatoprotection. Therefore, sea grapes are currently described not only as a daily food but also as a plant that can potentially have various therapeutic functions (12, 13). Sea grapes (*C. lentillifera*) have been widely studied for their role in improving lipid profiles, blood sugar, and cardiovascular and metabolic syndromes. A study by Preez et al. in Wistar rats fed with a high-carbohydrate and high-fat diet reported that the rats experienced a decrease in systolic blood pressure, body weight, plasma concentrations of total cholesterol, and non-esterified fatty acids, as well as a reduction in inflammation in liver tissue after being treated with *C. lentillifera* supplementation (12). This study provides insight that cardiometabolic risk factors can be reduced by supplementation with *C. lentillifera*, especially its ability to reduce inflammation and glucose metabolism, which are the keys to metabolic syndrome. Nguyen et al. reported that the ethanolic extracts of *C. lentillifera* have strong hydrogen peroxide scavenging activity, DPPH radical scavenging activity, ferric ion-reducing activity, and FIC activity (14). Moreover, sea grapes also contain various bioactive compounds such as bioactive peptides, dietary fiber, vitamins, minerals, polysaccharides, flavonoids, and polyunsaturated fatty acids (PUFA) (14). High in nutritional value, sea grapes are thought to be a functional food, especially for individuals with metabolic diseases such as type 2 diabetes, heart disease, hypertension, and the older adult population with high oxidative stress levels.

However, studies regarding the effect of *C. lentillifera* extract on serum glucose, lipid profile, PGC-1α levels, and oxidative capacity are still limited. Therefore, this study aims to assess the effect of sea grape (*C. lentillifera*) extract on serum glucose, lipid profile (total cholesterol), PGC-1α levels, and liver SOD levels in *Rattus novergicus* rats fed cholesterol-and fat-enriched diets (CFED) as primary outcomes. A change in the body weight of rats was reported as a secondary outcome.

## Materials and methods

### Production of sea grape extract

Fresh sea grapes (*Caulerpa lentillifera*) were collected in the shallows (5–10 m above sea level) of the Mantehage seawater, north of Sulawesi [coordinate google maps (1.7189753, 124.8034570)], Indonesia. Botanical identification and authentication were confirmed in the Department of Pharmacology, Faculty of Mathematics and Natural Sciences, Sam Ratulangi University, Indonesia. Specimens were collected for future reference. Sea grapes (whole-body) were thoroughly rinsed with water, dried at room temperature, baked at 40°C, and then ground with an electric grinder. Furthermore, in the extract preparation, coarse powder (1 kg) is macerated in 96% ethanol for 72 h, with each extraction carried out in triplicate, resulting in a yield of 34%. The extract is roughly filtered using Whatman 41 filter paper. The total filtrate is glued and evaporated at 40°C with the RV 8 IKA rotary evaporator under reduced pressure (100 millibars) for 90 min and evaporated in an oven at 40°C to produce the powder extract. The extract is stored in the refrigerator at a temperature of 4–8°C before being used in the experiment.

### Animal handling and ethical consent

All experimental rats were kept on standard free-feed and *ad libitum* water access. The research was conducted at the Pharmacology Laboratory, Faculty of Mathematics and Natural Sciences, Sam Ratulangi University, Manado, Indonesia. From the Animal Husbandry Laboratory of Makassar, Indonesia, forty male Wistar albino rats (*Rattus norvegicus*; 4–5 weeks) weighing 200–250 g each were obtained and transported to the study site. The minimum number of samples of Wistar albino rats were determined using the Federer formula, with the equation = (*t* – 1) (*r* – 1) ≥ 15, where *t* = treatment and *r* = replication. In this study, *t* is 4, then = (4 – 1) (*r* – 1) ≥ 15; 3(*r* – 1) ≥ 15; 3*r* – 3 ≥ 15; *r* ≥ 6. So the minimum sample for each group is 6. However, according to the recent review (15), we chose 10 samples per group to expect if there are samples that must be excluded.

The animals were grouped, housed in cages, and kept under standard laboratory conditions (temperature: 27 ± 2°C), with light and dark cycles (12/12 h). Rats were acclimatized to laboratory conditions for 10 days before the start of the experiment. The research protocol (the use of experimental animals) refers to the Declaration of Helsinki and the Council for International Organizations of Medical Sciences (CIOMS). In addition, all experimental procedures were performed in accordance with the Institutional Animal Care and Use Committee using the ARRIVE guidelines, the Ethics Committee of Faculty of Medicine, Sam Ratulangi University, and have been registered at Preclinical Trials Europe (www.preclinicaltrials.eu) with number PCTE0000264.

### An animal *in vivo* study design

#### Cholesterol- and fat-enriched diets production

Cholesterol- and fat-enriched diets (CFED) are standard rat foods containing 1% cholic acid, 2% pure cholesterol powder, 20% fat (animal source/pork oil), and 2% corn oil. Additional components were added finely to the standard CFED and homogenized into a dough by the addition of 1,000 ml of distilled water. Small pellets were cut and allowed to dry at room temperature under sterile conditions. CFED were prepared weekly and stored at 4°C until used to reduce oxidation. CFED consist of carbohydrates (43.57%), crude protein (12.38%), crude fiber (4.73%), crude fat (3.17%), cholesterol (2%), cholic acid (1%), animal fat (20%), corn oil (2%), total ash (4.3%), and moisture content (6.85%). Compared with a normal diet containing 58.1% carbohydrates, 16.51% crude protein, and 0% animal fat, all other components, such as corn oil, cholesterol, and folic acid, did not change significantly. CFED production guidelines were carried out as previously described (16).

#### The scheme of sea grape (*Caulerpa lentillifera*) extract administration

Albino male Wistar rats were randomly divided into four groups of ten each. Group A serves as control (receiving a standard dry pellet diet). Group B rats were fed CFED only for 4 weeks. Rats in Groups C and D were fed CFED and given sea grape extract 150 and 450 mg/kg BW for 4 weeks, respectively. We determined the doses based on the upper and lower capacity of the rat’s stomach. CFED and sea grape extract were administered orally.

#### Sample collection

Throughout the experiment, every effort was made to minimize the pain and suffering of the experimental animals. For this purpose, after 4 weeks of extract treatment, rats were put in fasting condition overnight and knocked out under an anesthetic of ketamine. As much as 2.5 ml of blood samples were collected from cardiac muscle tissue and stored in dry and clean tubes without the addition of anticoagulants (tiger-top tubes) to allow coagulation at room temperature. The sample was then centrifuged for 20 min at 3,000 rpm. Finally, serum was collected to analyze blood glucose, total cholesterol, and PGC-1α. Biomedical analysis of blood samples was done as follows: Blood glucose and cholesterol levels were tested using the COBAS Integra^®^ 400 plus analyzer (Roche). The sample was washed with 1% phosphate-buffered saline (PBS, pH 7.4) until the liquid was clear. Next, the sample was centrifuged at 3,000 rpm for 20 min to obtain pellets and supernatant. The supernatant was taken for PGC-1α assay. The concentration of PGC-1α was measured using a mouse PGC-1α ELISA Kit (Sunlong Biotech Co., Ltd., Hangzhou, China). SOD levels were calculated using the superoxide dismutase assay kit by Sigma-Aldrich with blood taken from liver tissue according to the product’s procedure kit.

### Data management and analysis

The data were statistically analyzed using the MANOVA/multivariate ANOVA test. The Levene test was used to determine which *post hoc* test should be performed. In cases where the *p*-value of Levene’s test was < 0.05, the Games-Howell test (equal variance was not assumed) was used, and for *p*-values > 0.05, the Bonferroni test (presumed equal variance) was used. Statistical analysis was performed using SPSS 26.0 for the Windows version.

## Results

### Characteristics of animal models

The characteristics of rats included in this study are shown in Table 1. Results indicated that CFED rats treated with sea grape extract exhibited a lower feed efficiency ratio compared to other groups. Subsequently, rats treated with sea grape extract yielded lower body weight changes relative to normal and CFED groups.

**TABLE 1:** Weight characteristics, food and water intake, and feed efficiency ratio between groups of experimental animals.

Groups	Body weight before treatment (g)	Body weight after treatment (g)	Body weight change (g/day)	Food Intake (g)	Water Intake (ml)	Feed efficiency ratio (FER, %)
Normal (A)	227.11 ± 15.46	257.53 ± 5.79	1.09 ± 0.64	5.29 ± 0.71	5.70 ± 0.73	21.22 ± 13.09
CFED (B)	227.49 ± 13.35	277.37 ± 7.22	1.78 ± 0.64	5.58 ± 0.40	5.60 ± 0.63	31.88 ± 11.48
CFED + 150 (C)	228.30 ± 12.30	239.70 ± 6.81	0.41 ± 0.34	5.85 ± 0.74	5.84 ± 0.40	7.24 ± 6.58
CFED + 450 (D)	224.62 ± 10.75	246.98 ± 5.63	0.80 ± 0.31	5.84 ± 0.56	5.83 ± 0.39	13.94 ± 6.07

CFED, cholesterol- and carbohydrates fat-enriched diets. Jeanette Irene Christiene Manoppo: JM Fahrul Nurkolis: FN Adriyan Pramono: AP Martha Ardiaria: MA Etisa Adi Murbawani: EM Muhammad Yusuf: MY Faqrizal Ria Qhabibi: FQ Vincentius Mario Yusuf: VY Nasim Amar: NA Muhammad Rico Abdul Karim: MK Anita Dominique Subali: AS Hans Natanael: HN Ronald Rompies: RR Rifrita Fransisca Halim: RH Alexander Sam Leonard Bolang: AB Gregory Joey: GJ Christian Agung Novianto: CN Happy Kurnia Permatasari: HP

### Blood glucose

**FIGURE 1:**
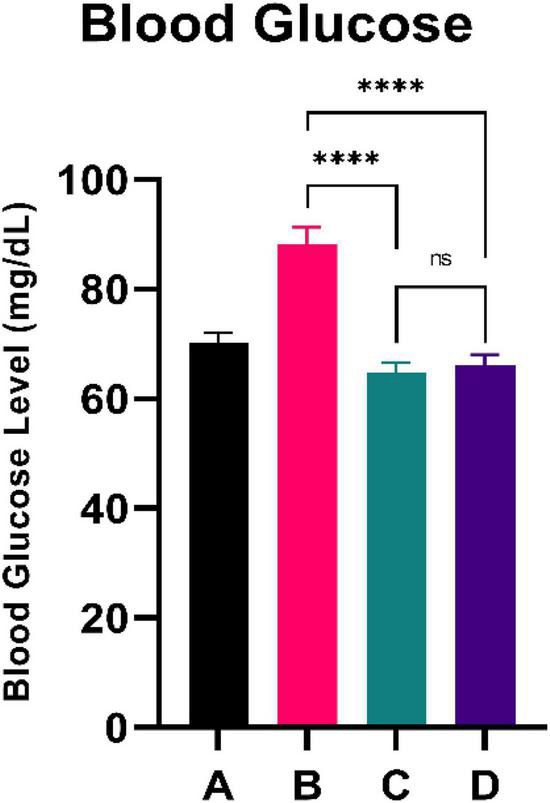
Low doses are more effective in lowering blood glucose. ****Means a strong *p*-value of < 0.0001.

Figure 1 illustrates the blood glucose level of the experimental rats. CFED rats gained significantly increased levels of blood glucose compared to the normal group. Furthermore, the two groups treated with sea grape extract showed a significantly lower blood glucose level than CFED rats. There was no significant difference in blood glucose between the 150 and 450 mg/kg BW sea grape extract groups.

### Total cholesterol

**FIGURE 2:**
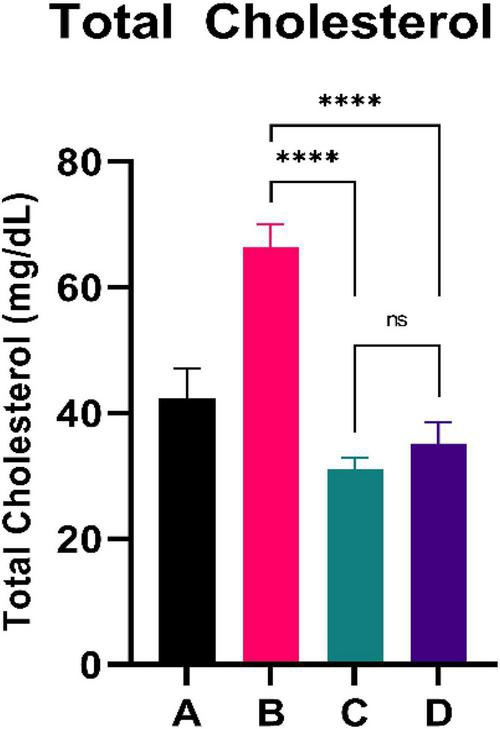
Low doses are more effective in lowering total cholesterol. ****Means a strong *p*-value of < 0.0001.

Figure 2 shows the total cholesterol level of experimental rats. CFED rats yielded significantly higher total cholesterol levels than the standard group. Furthermore, the two groups treated with sea grape extract showed a significantly lower total cholesterol level than CFED rats. There was no significant difference in total cholesterol between the 150 and 450 mg/kg BW of sea grape extract groups.

### PGC-1α

**FIGURE 3:**
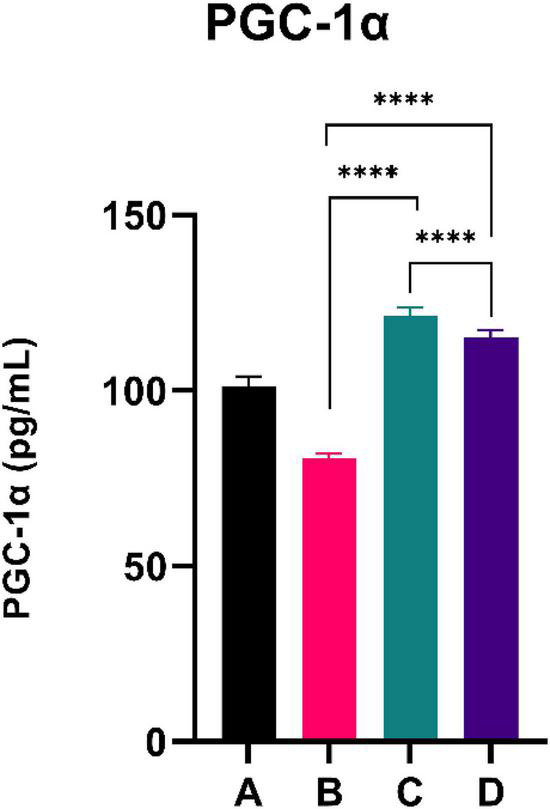
Low doses are more effective in improving the PGC-1a level. ****Means a strong *p*-value of < 0.0001.

The results of the PGC-1α level are shown in Figure 3. CFED rats exhibited a lower level of PGC-1a compared to the normal group. Moreover, the two groups treated with sea grape extract showed a significantly higher PGC-1α level than CFED rats. Interestingly, the treatment with 150 mg/kg BW of sea grape extract showed a higher increment of PGC-1α compared to the 450 mg/kg BW dose.

### Liver superoxide dismutase

**FIGURE 4:**
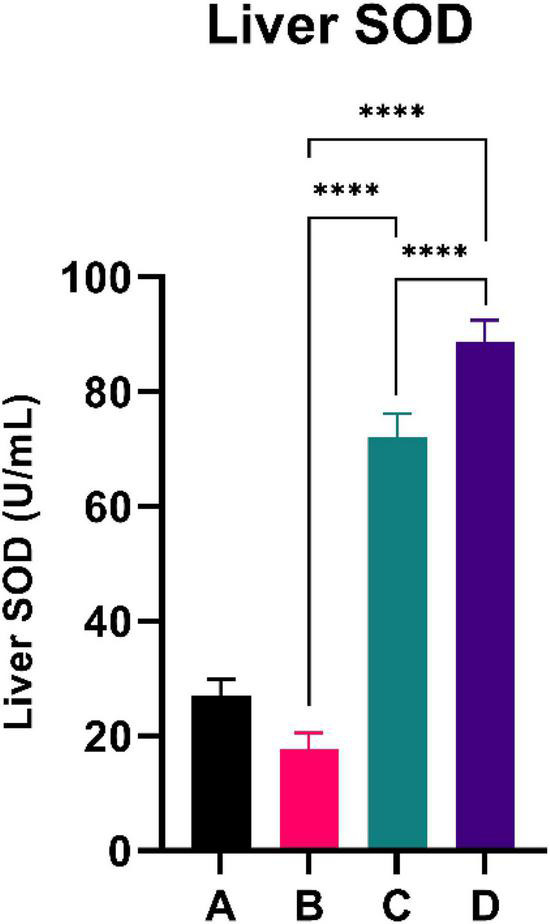
Both doses of sea grapes have a significant effect on liver SOD activity. ****Means a strong *p*-value of < 0.0001.

Figure 4 summarizes the PGC-1α level of the experimental rats. CFED rats exhibited a lower level of PGC-1a compared to the normal group. Interestingly, the two groups treated with sea grape extract showed a significantly higher PGC-1α level than CFED rats. The liver SOD level was higher with the 450 mg/kg BW dose of sea grape extract than with the 150 mg/kg BW dose. This suggests a dose-response manner toward the effect of sea grapes on liver SOD.

## Discussion

### The potential of sea grapes *(Caulerpa lentillifera)*

Seaweed has the potential to be cultivated as human food with its ample availability and the promising development of aquaculture, aside from fisheries (17). *C. lentillifera* is one of the green seaweeds that inhabit Southeast Asia and the Pacific seashore (18). It has a soft texture and palatable taste, and there are plenty of recipes for *C. lentillifera* as fresh vegetables (17). Indo-Pacific countries, such as the Philippines, Vietnam, and Japan, have cultivated *C. lentillifera.* Currently, *C. lentillifera* is the main product of commercial aquaculture (19, 20). Not only for its savory, *Caulerpa* species have gained popularity due to their availability, nutritional values, and general awareness as natural products (20). *C. lentillifera* contains sufficient dietary fibers, polysaccharides, essential unsaturated fatty acids, and protein (18). Moreover, recent research reveals potent beneficial bioactivity from *C. lentillifera.* Several studies have demonstrated that *C. lentillifera* has potent activity as an antidiabetic by improving insulin sensitivity (21, 22), regulating blood pressure (23), and exhibiting other health benefits such as anticancer as well as antimicrobial properties (24, 25). Thus, the development of *C. lentillifera* is relevant for human health since the prevalence of obesity-related disorders continues to rise.

### Safety aspect

Among the other Caulerpa species, *C. lentillifera* is considerably safer to consume because it does not accumulate toxic minerals found in the water it inhabits, such as arsenic, lead, and mercury (26). However, a recent study has suggested that the potential acidity of pond soil should be taken into account when cultivating Caulerpa to prevent toxic mineral accumulation in the water (27).

### Antioxidant property

Recently, antioxidant-rich foods have been attractive as part of healthy lifestyle trends in the protective role to counter reactive oxygen species (ROS) and other linked health conditions (28). ROS is remarkable for its ability to induce oxidative injury in human cells, which may lead to diverse chronic diseases, such as aging, cancer, and Alzheimer’s disease. Previous research demonstrated seaweed’s activity as a strong antioxidant to protect itself from ROS. The antioxidant properties are related to some phytochemical compounds, such as phenolics and flavonoids. These substrates have hydroxyl groups, which can donate hydrogen to stabilize free radicals and terminate new free radical generation (29). Oxidative stress has been suggested to link with obesity and induces metabolic syndrome (6).

### Possible mechanisms to alter the metabolic profile in metabolic syndrome

Recent research by Preez et al. on rats with a high-carbohydrate and high-fat (HCHF) diet exhibited *C. lentillifera*’s supplementation ability for improving cardiometabolic risk factors. The study showed that feeding rats with such a diet established hypertension, dyslipidemia, fatty liver disease, obesity, and increased collagen deposition inside the left ventricle (12). *C. lentillifera-*augmented diet in HCL rats displayed positive effects by reducing some parameters, such as body weight, systolic blood pressure, plasma concentrations of total cholesterol and non-esterified fatty acids, heart and liver inflammatory cells, and visceral adiposity (12).

Another study also displayed a potential modulation in the gut microbiota by *C. lentillifera*. Supplementation of *C. lentillifera* decreased the Firmicutes to Bacteroidetes ratio. Some possible mechanisms explain green algae’s health benefits, such as prebiotic effects due to their high fiber content. In *Caulerpa* species, sulfated polysaccharides have complex and heterogeneous repetitive sugar unit structures. Polysaccharides of *Caulerpa* sp. are digested minimally in the stomach but will be further fermented by bacteria residing in the colon as prebiotics (30). It has been shown that prebiotics from the ocean biodiversities gives impacts on metabolic health, such as reducing body weight and blood pressure (31). Another study on diet-induced obesity discovered that inulin and oligofructose-mixed prebiotic is an effective dietary fiber to reduce body weight gain, systolic blood pressure, plasma concentrations of triglycerides, and free fatty acids and attenuate inflammatory cells to infiltrate the heart and the liver (32).

The insoluble fiber in *C. lentillifera* is proposed to be linked with enhanced short-chain fatty acids (SCFAs) production inside the colon, such as butyric, propionic, and acetic acids (33). Most of them are insoluble fibers, which are not converted into energy and enhance satiety (34). Escalating soluble fiber intake with inulin and oligofructose has exhibited improved metabolic syndrome signs by abating gastrointestinal uptake of carbohydrates and lipids (33). Polysaccharides from ocean biodiversities work with diverse mechanisms through selective fermentation, gut pH lowering, fecal bulking, gut pathogen colonization prevention, and putrefactive bacterial control. As a result, they can protect the host from toxic metabolite exposure (35). These outcomes may be related to the activity of dietary fiber to increase SCFA production, providing energy to the host (36). Unfortunately, in our *in vivo* model, we were unable to analyze the gut microbiota composition and SCFAs. Therefore, it could be recognized as a limitation of our study.

Since obesity and metabolic syndrome are often characterized by chronic low-grade inflammation (1), reducing the proinflammatory state is vital to preventing other metabolic disorders. One possible strategy is by consuming food that contains compounds with anti-inflammatory activity. This functional type may help to reduce systemic chronic low-grade inflammation in obesity (37). Polysaccharides of *C. lentillifera* can enhance immunostimulatory and anti-inflammatory activity (38), which may improve antioxidant capacity (i.e., increases SOD capacity) in the liver.

This experimental study reported that two groups of rats with the intervention of sea grape extract (150 and 450 mg/kg BW) had significantly lower blood glucose levels than the CFED rat group (Figure 1). This result is in line with the studies by Permatasari et al. (16) and Kuswari et al. (39), which also showed a significant decrease in blood glucose levels in CFED rats after being treated with sea grape extract. CFED feed in rats can significantly increase blood glucose levels and lipid profiles. We also found a significant decrease in total cholesterol levels in the group of rats given CFED + sea grape extract compared to CFED-only rats (Figure 2). However, the results of lipid profile and blood glucose level improvements between 2 groups of rats with sea grape extract doses of 150 and 450 mg/kg BW showed no significant difference. Interestingly, study results found no significant difference in total cholesterol between the high dose and low dose group; this might be due to palmitate acid, which dominates fatty acid composition in sea grapes, being able to raise total cholesterol levels in the blood (40). In contrast, the group of rats treated with the sea grapes had higher levels of PGC-1 alpha than the group of CFED rats (Figure 3). An increase in PGC-1 alpha is essential for the regulation of cellular energy metabolism (Graphical abstract). In addition, PGC-1 alpha is also expressed in tissues with high energy demand and is strongly associated with the occurrence of metabolic syndrome. Interestingly, this study reported that the group of rats treated with a dose of 150 mg/kg BW sea grapes had higher levels of PGC-1 alpha compared to a dose of 450 mg/kg BW. Furthermore, it was found that the effect of sea grapes on liver SOD levels was dose-dependent; the higher the dose, the higher the liver SOD levels (Figure 4). There was no significant difference in body weight between all groups of rats.

*C. lentillifera* contains various bioactive molecules that can contribute to its anti-hyperglycemic activity, including sulfated polysaccharides and monosaccharides. A previous study by Fajriah et al. showed that purified polysaccharides of *C. lentillifera* had significant inhibition capability against α-glucosidase (41). A -glucosidase is an enzyme located in the brush borders of the small intestine, which operates by cleaving disaccharides into glucose to be further absorbed (42). Inhibiting α-glucosidase can delay glucose uptake and reduce sugar circulating in the bloodstream. This proposed mechanism is similar to present oral antidiabetic drugs for type 2 diabetes, such as acarbose and miglitol, used in clinical practice (43). In addition, a study by Sharma et al. showed that *C. lentillifera* extract significantly increased insulin secretion, glucose transporter expression, and enhanced glucose uptake in adipocyte cells *in vitro* (22). Furthermore, in a study, *C. lentillifera* also significantly decreased the DPP-4 enzyme, which increased circulating incretin levels, leading to increased insulin release and improved glycemic control (21).

This study showed that the administration of *C. lentillifera* resulted in a significant reduction in cholesterol (Graphical abstract). A possible mechanism is that *C. lentillifera* contains caulerpenyne, a major metabolite of the *Caulerpa* genus in the form of sesquiterpenoids that are shown to inhibit lipase activity *in vivo* competitively (44). Moreover, this lipase-inhibitory activity of *C. lentillifera* might also be attributed to its high phenolic content, especially flavonoids (29, 45, 46). Inhibition of lipase causes reduced lipolysis of dietary fats entering the digestive tract, decreasing the amount of fatty acid taken into the bloodstream, which eventually reduces the formation of LDL, VLDL, and TAG, the main components of total cholesterol in the liver. An increased level of SOD (potentially reduced oxidative stress) in the liver may also partly contribute to improving liver lipid metabolism (8). The mentioned mechanism may also cause a decrease in post-intervention body weight of experimental animals due to less ectopic fat (4).

Next, systemic PGC-1α, the most well-known and studied member of transcriptional coactivators called the PGC-1 family, has increased significantly after *C. lentillifera* administration. PGC-1α influences most cellular metabolic pathways and plays an essential role in mitochondrial biogenesis, especially in detoxifying reactive oxygen species by regulating the expression of ROS-detoxifying enzymes (6). The PGC-1α activation pathways can upregulate the expression of Sirtuin 1 (SIRT1), antioxidants, including glutathione peroxidase and SOD, which may be further demonstrated in this study as liver SOD increased significantly in rats post-intervention (47). Antioxidants are crucial to scavenge ROS in metabolic disorders since excess ROS are involved in insulin signal dysregulation, insulin resistance, overfeeding, saturated fatty acids, and chronic inflammation (48). Deregulation and decrease in the PGC-1α expression itself have also been linked strongly to trigger various metabolic disorders, including obesity, cardiovascular diseases, and NAFLD that cause various inflammatory processes with dysfunctional redox control; therefore, PGC-1α modulation is significant to provide clinical metabolic benefits (47, 48) potentially. Summarized mechanisms of *C. lentillifera* can be seen in Graphical abstract.

In general, *C. lentillifera* has promising potential as a prospective nutraceutical for patients with obesity-induced metabolic disorders, including the improvement of hyperglycemia in diabetic patients, the suppression of hyperlipidemia, hypercholesterolemia, and the reduction in weight in patients with obesity. The increase in PGC-1α also contributes to various and vast positive impacts on oxidative metabolism, which can benefit patients with metabolic disorders.

## Conclusion

Sea grape (*C. lentillifera*) extract showed potential efficacy as nutraceuticals in improving blood glucose, total cholesterol, PGC-1α, and liver SOD levels in rats that were fed CFED. Providing a dose of 150 mg/kg of BW effectively lowers blood glucose and total cholesterol by increasing PGC-1α levels. The dose obtained from preclinical trials could be a reference for future clinical trials in humans.

## Data availability statement

The raw data supporting the conclusions of this article will be made available by the authors, without undue reservation.

## Ethics statement

The animal study was reviewed and approved by the research protocol or use of experimental animals refers to the Declaration of Helsinki with the Council for International Organizations of Medical Sciences (CIOMS). In addition, this research protocol is performed according to the Institutional Animal Care and Use Committee using the ARRIVE Guidelines, Ethics Committee of Faculty of Medicine, Sam Ratulangi University and has been registered at Preclinical Trials Europe (www.preclinicaltrials.eu) with number PCTE0000264.

## Author contributions

JM and FN conducted experiments, analyzed data, wrote manuscripts, and conceptualized and designed the study. MY, FQ, VMY, NA, MK, AS, HN, RR, RH, AB, GJ, CN, HP, AP, MA, and EM contributed to data analysis, data interpretation (AP), wrote manuscripts, interpret results, and editing. All authors have read and also approved this final manuscript.

## Abbreviations

BW: body weight
CFED: cholesterol- and fat-enriched diets
CIOMS: Council for International Organizations of Medical Sciences
DPP-4 enzyme: dipeptidyl peptidase-4
DPPH: 2,2-diphenyl-1-picrylhydrazyl
FIC: ferric ion reducing
HCL: hydrochloric acid
IL-12: interleukin-12
IL-1β: interleukin-1 beta
LDL: low-density lipoproteins
PCG-1α: peroxisome proliferator-activated receptor-gamma coactivator (PGC)-1 alpha
ROS: reactive oxygen species
SCFAs: short chain fatty acids
SIRT1: sirtuin-1
SOD: superoxide dismutase
TG: triacylglycerol
TNF-α: tumor necrosis factor alpha
VLDL: very low-density lipoproteins.
